# Optimization of Hot Rolling Scheduling of Steel Strip with High Bending Performance

**DOI:** 10.3390/ma15041534

**Published:** 2022-02-18

**Authors:** Chien-Cheng Feng, Ming-Hong Lin, Wei-Heng Chuang, Yi-Cheng Chen, Shih-Fu Ou

**Affiliations:** 1 Department of Mechanical Engineering, National Kaohsiung University of Science and Technology, No. 415, Jiangong Rd., Sanmin Dist., Kaohsiung 80778, Taiwan; I109142106@nkust.edu.tw (C.-C.F.); mhlin@nkust.edu.tw (M.-H.L.); J107242119@nkust.edu.tw (W.-H.C.); 2 Department of Mold and Die Engineering, National Kaohsiung University of Science and Technology, No. 415, Jiangong Rd., Sanmin Dist., Kaohsiung 80778, Taiwan; smn874022000@gmail.com

**Keywords:** hot-rolled steel, pearlite-banded structures, Taguchi method

## Abstract

Poor formability in hot-rolled strips may be attributed to the many pearlite-banded structures (PBSs) that develop in steel during the hot-rolling process. The challenge of manufacturing strips with minimum PBSs is that multiple factors influence the amount and distribution of the PBSs. This study used the Taguchi method to find the optimum hot-rolling parameters to obtain strips with a reduced number of PBSs. The strips were then subjected to bending tests to evaluate their ductility. The first part analyzes the contribution of selected parameters to the hot-rolling process: (1) finishing rolling temperature, (2) finishing rolling speed, (3) coiling temperature, and (4) coiling speed. The second part confirms, using bending tests, the influence of the finishing rolling temperatures 780, 800, 820, 840, 860, 870, and 880 °C on the formability of an A36 hot-rolled strip. Based on the experimental protocol for the study, the optimal process parameters were determined to be the finishing rolling speed (0.80 m/s), finishing rolling temperature (870 °C), coiling speed (2.80 m/s), and coiling temperature (650 °C). When the A36 strip was prepared at the optimum parameters, the average length and thickness of the PBS were 108.61 ± 0.11 μm and 10.18 ± 0.12 μm, respectively. According to the Taguchi analysis, the finishing rolling temperature had the most significant influence on the dimensions of the PBS. In tests where the hot-rolled A36 strip was bent to 90° and 180°, at the finishing rolling temperatures of 870 °C and 880 °C, no cracking was observed at the R angle.

## 1. Introduction

The microstructural banding of pearlite, ferrite, bainite, and martensite arranged in layers has commonly been observed in hot-rolled steel strips [1]. The microstructural banding in hypoeutectoid steels can be attributed to two kinds of segregations [2,3,4,5]. One is original segregation from the continuous casting of the slab. The other is microsegregation which is induced by phase transformation during cooling. The addition of manganese in low-alloy steels causes dendritic solidification, comprising the dendrite heart and the interdendritic spaces with a high and low-manganese amount, respectively. The distribution of manganese dominates the transformation temperature of austenite to ferrite (Ar_3_ temperature). In the cooling process, a carbon atom migrates from the low-manganese regions toward the high-manganese regions and therefore, the carbon-rich and carbon-depleted regions form ferrite and perlite layers, respectively. The microstructural banding causes anisotropy in the tensile ductility and impact energy of the steels [6]. Bor indicates that the microstructural banding has an adverse effect on the ductility of the steel evaluated by impact tests [7]. The presence of pearlite-banded structures (PBSs) negatively affects the formability of hot-rolled steel, leading to a decrease in the impact toughness, transverse plastic toughness, and fracture toughness of steel [1,8,9]. Coarse PBS causes the cracking and uneven deformation of steel, which becomes bent or deep drawn, respectively.

In recent years, researchers have made numerous attempts to improve the quality of hot-rolled steel. Research has indicated that homogenization can reduce or eliminate the micro-segregated banded ferrite and pearlite structures. However, the steel must be heated to a high temperature in a furnace over a long time before it is thoroughly homogenized. Hence, this method is expensive and time-consuming.

In addition, the annealing of hot-rolled steel with an increased cooling rate has also been proposed. Theoretically, if the annealed steel is cooled more rapidly than the pearlite-precipitation rate, the formation of PBSs will be inhibited [10,11]. Adjusting the hot-rolling process to minimize the amount of PBSs formed is the most economical way to improve the fracture toughness of steel [12,13]. However, other relatively complex parameters of the hot-rolling process, such as the moving velocity and temperature, are less discussed in related literature.

This study used the Taguchi method to find the optimum hot-rolling parameters to obtain steel with a reduced amount of PBSs. The variables investigated include: (1) finishing rolling temperature, (2) finishing rolling speed, (3) coiling temperature, and (4) coiling speed. In addition, the contribution of the parameters to the PBS size was analyzed.

## 2. Materials and Methods

This study used a commercially available hot-rolled slab (A36) manufactured using low-carbon steel with the chemical composition (wt.%): 0.12 C, 0.31 Si, 0.9 Mn, 0.01 P, and 0.01 S, and bal. Fe. The slab’s lower yield stress, tensile strength, and elongation were 324 MPa, 457 MPa, and 33%, respectively.

**Figure 1:**
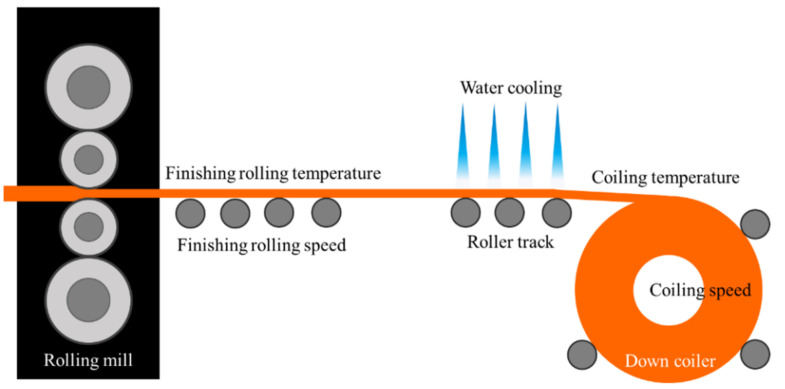
Schematic illustration of the hot rolling process.

The A36 slab was annealed at 1150 °C to obtain an austenitic microstructure. After cleaning the surface, the slab was rolled from 175 to 28 mm and then rolled further to achieve a strip thickness of 12 mm. The schematic illustration of the hot rolling process is shown in Figure 1.

To investigate the effects of the hot-rolling process on the distribution of the PBSs in A36 strips, the Taguchi method was used to design the experimental parameters. Four parameters were considered: (1) finishing rolling speed, (2) finishing rolling temperature, (3) coiling speed, and (4) coiling temperature. Table 1 lists the different variations of the four parameters using an L9 Taguchi orthogonal configuration [14].

The microstructures of the resultant A36 strips were observed via optical microscopy (OM, OLYMPUS PMG3) after polishing and subsequent etching with a 3% Nital solution. The average thickness and length of the PBS in the strips were obtained through measurement at three sites in a OM image, and three OM images were used for this purpose. In the OM image, the top three largest thicknesses and lengths of the PBS among the pearlite layers were measured for calculating the average value with standard deviation. Variance analysis (ANOVA) was performed to determine the influence of the factors on the PBS size. The signal-to-noise (S/N) ratio was used to optimize the responses of the variables. In the S/N ratio, signal refers to the real value which is desired, and noise refers to the undesired factors in measured values. There are three fundamental classifications to decide the best consequences of experiments; the smaller-the better-characteristic formulas used in this study are given below:

$$S\;/{\;N}_{i}=-10\log_{10}\left[\frac{1}{n}\sum_{i=1}^{n}y_{i}{}^{2}\right]$$

where *n* is the number of measurements, and y*_i_* is the *i*th observation. The unit of S/N ratio is dB (decibel).

Furthermore, to confirm the optimal parameters for the hot-rolling process, bending tests were performed on the resultant A36 strips at a bending speed of 1 mm/s using a CS-60062 bending machine (Chen Sheng Industry Co., Taoyuan City, Taiwan). The strips were bent to 90° and 180° and then analyzed for crack formation using OM.

## 3. Results and Discussion

### 3.1. Single-Target Quality Parameter Optimization of the Hot-Rolling Process

**Figure 2:**
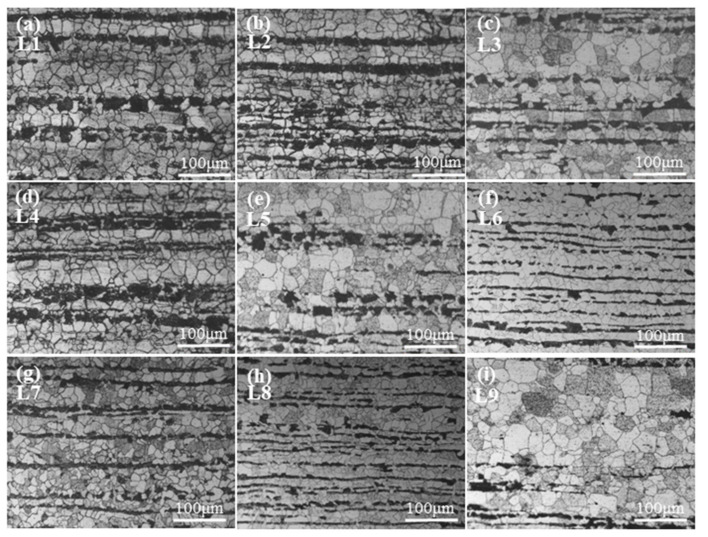
OM images of (**a**) L1, (**b**) L2, (**c**) L3, (**d**) L4, (**e**) L5, (**f**) L6, (**g**) L7, (**h**) L8, and (**i**) L9 samples.

**Figure 3:**
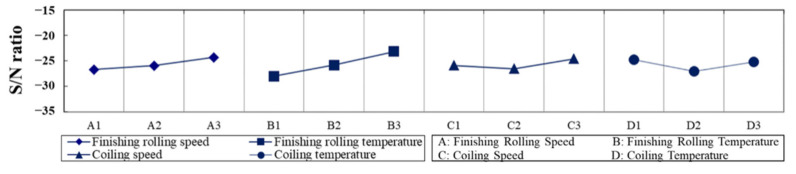
Main effect plot for S/N ratios of the thickness of PBSs.

OM images of the samples presented in Figure 2 show that their microstructures consist of white ferrite and black perlite grains. The distribution of the PBS in the rolling direction was analyzed. For L1, L2, L4, L6, L7, and L8, the PBS were continuously distributed and of a higher thickness than L3, L5, and L9. Table 2 and Figure 3 provide data on PBS thickness measurements and S/N ratio calculations. Table 3 shows the contributions of the four factors that were varied in the investigation. The results indicate that the general order of significance for the four factors is B > D > A > C.

**Figure 4:**
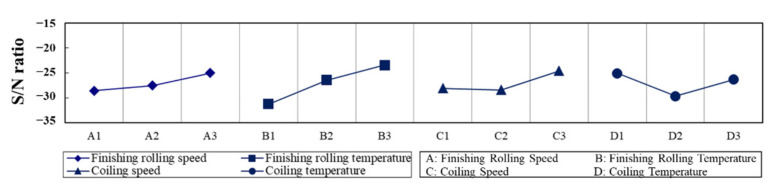
Main effect plot for S/N ratios of the length of PBSs.

The optimum thickness of the PBS was approximately 10.18 μm, which was fabricated using the parameter combination A3B3C3D1, i.e., a finishing rolling temperature of 870 °C, a finishing rolling speed of 0.80 m/s, a coiling temperature of 650 °C, and a coiling speed of 2.80 m/s. Table 4 lists the PBS lengths and the calculated S/N ratios, as shown in Figure 4. The order of the factors influencing the PBS length was determined to be B > D > C > A (Table 5), and the parameter combination A3B3C3D1 produced steel with the shortest PBS length (~108.61 ± 0.11 μm) and the least PBS thickness (~10.18 ± 0.12 μm).

### 3.2. The Effect of Hot-Rolling Parameters on PBS Thickness and Length

**Figure 5:**
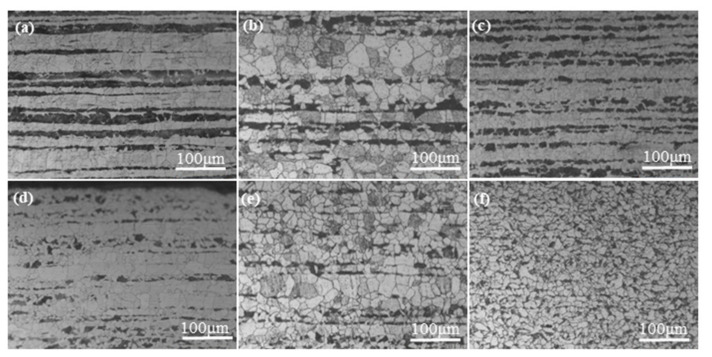
OM images of A36 strips fabricated under A3C3D1 conditions and the finishing rolling temperatures (**a**) 780, (**b**) 800, (**c**) 820, (**d**) 840, (**e**) 860, and (**f**) 880 °C.

According to the Taguchi analysis, the finishing rolling temperature had the most significant influence on PBS distribution. This section of the study used the optimal parameter combination A3C3D1 with six finishing rolling temperatures (780, 800, 820, 840, 860, and 880 °C) to hot-roll the A36 slab, and OM was used to observe the samples. From OM images (Figure 5), the PBS thickness decreased, and the structure became discontinuous when the finishing rolling temperature was raised.

**Figure 6:**
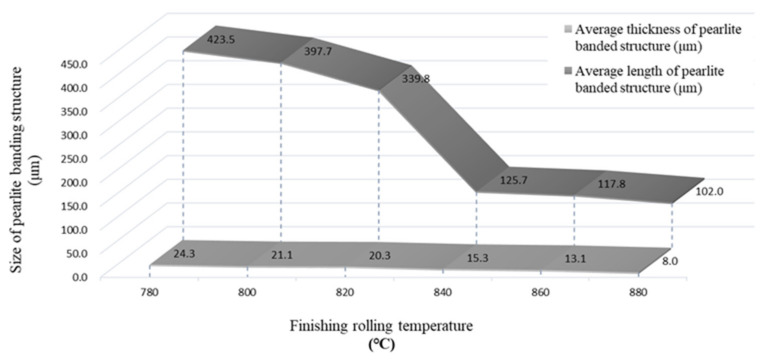
Influence of different finishing rolling temperatures on the average PBS thickness and length.

The measured thickness and length of the PBSs are listed in Table 6 and Table 7 and they are plotted in Figure 6. The PBS size decreased with the increasing finishing temperature, especially when the temperature was above 840 °C. This decrease is related to the compression and elongation of austenite grains during the hot-rolling process at temperatures above the recrystallization temperature of the steel. Therefore, there is significant dislocation accumulation above a finishing temperature of 840 °C, leading to the formation of discontinuous bands [15,16].

The accumulation of the strain energy can also transform austenite into ferrite; this is equivalent to increasing the Ar_3_ temperature, shortening the precipitation time of the eutectoid ferrite, and reducing the PBS size [17]. In addition, the PBS size decreased with raising the finishing rolling speed and the hot-rolling and coiling speed. As a result of the rapid cooling process after hot-rolling, carbon does not have enough time to diffuse in the steel [1,10,13,18,19]. Thus, austenite tends to transform into ferrite, and the PBS size is relatively small. In the report from Samuels, the PBS appeared when austenite was cooled at a relatively low cooling rate. However, the PBS was not observed when the cooling rate was increased to a range of 1500~3000 °C/h [18].

### 3.3. Verification of the Bending Test

**Figure 7:**
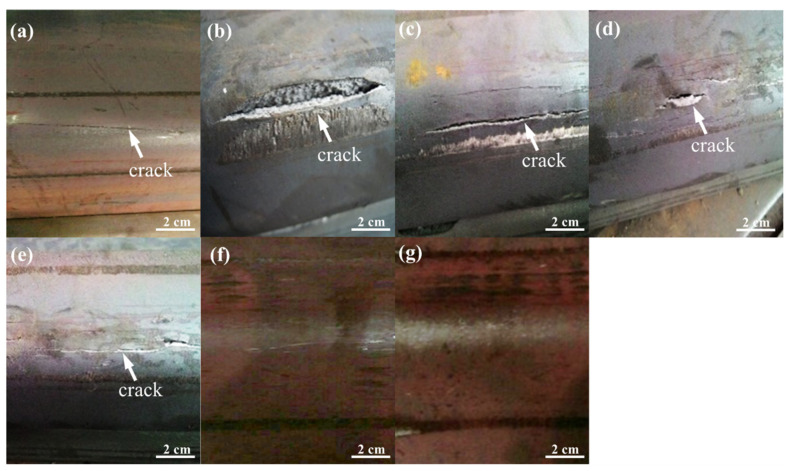
Hot-rolled A36 strips bent to 90° at the finishing rolling temperatures (**a**) 780, (**b**) 800, (**c**) 820, (**d**) 840, (**e**) 860, (**f**) 870, and (**g**) 880 °C.

Figure 7 shows A36 strips fabricated using A3C3D1 at different finishing rolling temperatures after bending tests. Cracks were observed on the surface in the bending region of the strips for the finishing rolling temperatures 780, 800, 820, 840 and 860 °C (Figure 7a–e). However, no cracks were observed while bending the strips at 870, and 880 °C, as shown in Figure 7f,g, respectively. The formation of the cracks can be divided into three distinct stages; in the first stage, shear micro-cracks are formed in the PBS; in the second stage, the crack extends to the adjacent ferrite grain; in the third stage, the crack propagates to the boundary of the ferrite grains [20,21,22]. Hence, the hot-rolled strip with an 860 °C finishing rolling temperature which was bent to 90°, still exhibited small cracks (Figure 7e) due to the appearance of a few PBSs in the grains (Figure 5e).

**Figure 8:**
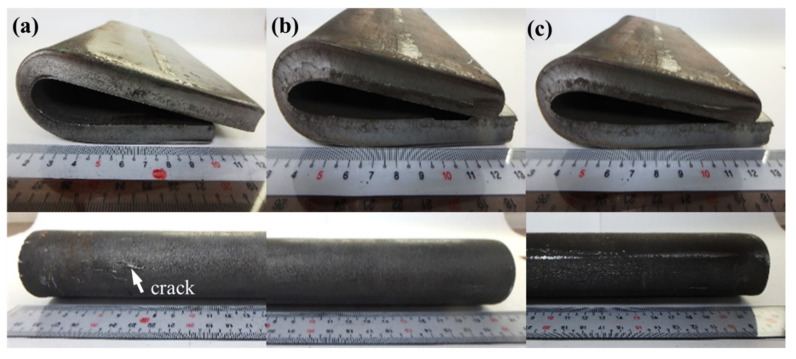
Hot-rolled A36 strips bent to 180° at the finishing rolling temperatures (**a**) 860, (**b**) 870, and (**c**) 880 °C.

Furthermore, at the finishing rolling temperatures 870 and 880 °C, the A36 strips were bent to an angle of 180°, yielding the results shown in Figure 8. Cracks were observed on the surfaces of the bent sides at 860 °C (Figure 8a), but were not observed at 870 and 880 °C (Figure 8b,c). This observation implies that reducing the PBS size significantly improves the bending performance of the A36 strips. Furthermore, related literature indicates that reducing the PBS size can reduce the depth of the local corrosion of ferrite-pearlite steel in a chloride environment [23].

## 4. Conclusions

This study used the L9 Taguchi method (3^4^) to design different process parameters to hot-roll an A36 slab and discussed their influence on the thickness and length of the PBS. Following this, A36 strips fabricated using the optimum parameters were analyzed by bending tests at different temperatures. The conclusions from the study can be summarized as follows:

1. The order of the influence of the hot-rolling process parameters on the PBS thickness was (B) finishing rolling temperature > (A) finishing rolling speed > (D) coil temperature > (C) coiling speed; the optimum parameter combination was determined to be A3B3C3D1, corresponding to a finishing rolling temperature of 870 °C, a finishing rolling speed of 0.80 m/s, a coiling temperature of 650 °C, and a coiling speed of 2.80 m/s. Under these conditions, the optimum PBS thickness of the hot-rolled A36 strips was approximately 10.18 ± 0.12 μm.
2. The order of influence of the hot-rolling process parameters on the PBS length was (B) finishing rolling temperature > (D) coil temperature > (C) coiling speed > (A) finishing rolling speed. The optimum parameter conditions A3B3C3D1 produced hot-rolled A36 strips with a PBS length of approximately 108.61 ± 0.11 μm.
3. As the finishing rolling temperature increased, the PBS thickness and length of the hot-rolled strip decreased. Furthermore, when the finishing rolling temperature was 870 and 880 °C, cracks were not observed on the surface of the bending regions of the A36 strips for the bending angles 90° and 180°.

## Figures and Tables

**Table 1:** Tabulation of levels according to L9 orthogonal array.

	A	B	C	D
Experiment Number	Finishing Rolling Speed (m/s)	Finishing Rolling Temperature (°C)	Coiling Speed (m/s)	Coiling Temperature (°C)
1	0.60	810	2.20	650
2	0.60	840	2.50	700
3	0.60	870	2.80	750
4	0.70	810	2.50	750
5	0.70	840	2.80	650
6	0.70	870	2.20	700
7	0.80	810	2.80	700
8	0.80	840	2.20	750
9	0.80	870	2.50	650

**Table 2:** Thickness of PBSs per sample and their average value, standard deviation, and S/N ratios for the 9 experiments.

Experiment Number	Samples			Average Value	Standard Deviation	S/N
	1	2	3			
1	25.66	25.83	26.97	26.15	0.71	−28.35
2	25.64	28.99	31.12	28.58	2.76	−29.16
3	13.00	13.54	13.88	13.47	0.44	−22.59
4	25.41	26.74	28.96	27.04	1.79	−28.66
5	14.33	15.89	17.51	15.91	1.59	−24.08
6	16.32	18.21	19.14	17.89	1.44	−25.08
7	21.37	22.80	23.00	22.39	0.89	−27.01
8	15.61	16.22	16.87	16.23	0.63	−24.21
9	11.89	12.02	12.52	12.14	0.33	−21.69
Average value				19.98	1.18	−25.65

**Table 3:** ANOVA results for the thickness of PBSs.

Factor	SS	DOF	Var	Contribution
A	9.013	2	4.506	14.9%
B	35.926	2	17.963	59.5%
C	5.916	2	2.958	9.8%
D	9.567	2	4.784	15.8%
Total	60.422	8	-	100%

**Table 4:** Length of PBSs per sample and their average value, standard deviation, and corresponding S/N ratios for the 9 experiments.

Experiment Number	Samples			Average Value	S	S/N
	1	2	3			
1	392.61	393.51	393.98	393.37	0.70	−51.90
2	396.21	397.53	397.85	397.20	0.87	−51.98
3	122.69	123.65	123.96	123.43	0.66	−41.83
4	417.35	419.67	420.00	419.01	1.44	−52.44
5	132.38	133.20	135.81	133.80	1.79	−42.53
6	240.28	241.55	242.36	241.40	1.05	−47.65
7	295.20	298.33	298.98	297.50	2.02	−49.47
8	172.33	173.94	175.61	173.96	1.64	−44.81
9	109.28	110.32	110.94	110.18	0.84	−40.84
Average value				254.43	1.22	−47.05

**Table 5:** ANOVA results for the length of PBSs.

Factor	SS	DOF	Var	Contribution
A	19.759	2	9.879	11.3%
B	93.602	2	46.801	53.7%
C	26.954	2	13.477	15.5%
D	34.050	2	17.025	19.5%
Total	174.365	8	-	100%

**Table 6:** The thickness of PBS measured from three images of A36 strips fabricated under A3C3D1 condition with various finishing rolling temperatures.

Finishing Rolling Temperature (°C)	Image 1			Image 2			Image 3			Average PBS Thickness (μm)
	Site			Site			Site			
	1	2	3	1	2	3	1	2	3	
780	28.7	28.9	26.8	22.3	21.7	24.3	21.4	22.3	21.9	24.3 ± 3.1
800	20.7	21.1	20.8	21.3	21.4	21.2	21.2	21.4	20.9	21.1 ± 0.3
820	20.2	20.9	20.5	19.6	20.9	19.5	20.9	20.1	19.9	20.3 ± 0.6
840	15.7	14.9	15.3	15.2	15.8	14.7	14.9	15.5	15.4	15.3 ± 0.4
860	13.1	12.9	12.9	13.3	13.2	13.0	13.2	13.1	13.0	13.1 ± 0.1
880	8.4	8.1	8.0	8.2	8.0	7.8	8.1	7.9	7.8	8.0 ± 0.2

**Table 7:** The length of PBS measured from three images of A36 strips fabricated under A3C3D1 condition with various finishing rolling temperatures.

Finishing Rolling Temperature (°C)	Image 1			Image 2			Image 3			Average PBS Length (μm)
	Site			Site			Site			
	1	2	3	1	2	3	1	2	3	
780	422.4	420.9	416.7	435.0	433.0	420.2	432.1	420.6	410.6	423.5 ± 8.2
800	404.1	394.6	392.3	402.1	400.0	396.4	399.7	398.2	392.3	397.7 ± 4.2
820	342.2	339.3	337.7	345.0	343.3	338.9	343.3	338.9	329.4	339.8 ± 4.6
840	127.1	124.5	124.3	129.3	125.6	124.9	126.7	124.7	124.3	125.7 ± 1.7
860	120.9	117.3	116.9	118.0	116.6	115.9	118.5	118.2	117.9	117.8 ± 1.4
880	104.3	104.3	103.8	104.3	101.6	100.1	100.1	99.9	99.2	102.0 ± 2.2

## Data Availability

Data sharing is not applicable to this article.
